# Investigation of Differences in Fertility among Progenies from Self-Pollinated Chrysanthemum

**DOI:** 10.3390/ijms19030832

**Published:** 2018-03-13

**Authors:** Fan Wang, Xinghua Zhong, Haibin Wang, Aiping Song, Fadi Chen, Weimin Fang, Jiafu Jiang, Nianjun Teng

**Affiliations:** College of Horticulture, Nanjing Agricultural University, Key Laboratory of Landscape Agriculture, Ministry of Agriculture, Nanjing 210095, China; 2015204035@njau.edu.cn (F.W.); xinghua_92@163.com (X.Z.); hb@njau.edu.cn (H.W.); aiping_song@njau.edu.cn (A.S.); chenfd@njau.edu.cn (F.C.); fangwm@njau.edu.cn (W.F.); jiangjiafu@njau.edu.cn (J.J.)

**Keywords:** *Chrysanthemum morifolium*, self-incompatibility, seed set, RNA-seq, *S* genes

## Abstract

Most chrysanthemum cultivars are self-incompatible, so it is very difficult to create pure lines that are important in chrysanthemum breeding and theoretical studies. In our previous study, we obtained a self-compatible chrysanthemum cultivar and its self-pollinated seed set was 56.50%. It was interesting that the seed set of its ten progenies ranged from 0% to 37.23%. Examination of the factors causing the differences in the seed set will lead to an improved understanding of chrysanthemum self-incompatibility, and provide valuable information for creating pure lines. Pollen morphology, pollen germination percentage, pistil receptivity and embryo development were investigated using the in vitro culture method, the paraffin section technique, scanning electron microscopy and transmission electron microscopy. Moreover, RNA sequencing and bioinformatics were applied to analyzing the transcriptomic profiles of mature stigmas and anthers. It was found that the self-pollinated seed set of “Q10-33-1①”,”Q10-33-1③”,”Q10-33-1④” and “Q10-33-1⑩” were 37.23%, 26.77%, 7.97% and 0%, respectively. The differences in fertility among four progenies were mainly attributable to differences in pollen germination percentage and pistil receptivity. Failure of the seed set in “Q10-33-1⑩” was possibly due to self-incompatibility. In the transcriptomic files, 22 potential stigma *S* genes and 8 potential pollen *S* genes were found out.

## 1. Introduction

The cultivated chrysanthemum is generally allohexaploid and aneuploidy [1,2] with a highly heterozygous genotype, and its trait inheritance and genetic background are extremely complex [3,4] so it is very hard to create chrysanthemum pure lines. However, chrysanthemum pure lines are very important for the utilization of heterosis and genome research. Faced with this, self-fertilization is an important means to develop chrysanthemum pure lines. Usually, pure lines can be created by the multigenerational selfing of self-compatible chrysanthemum cultivars. However, most cultivated chrysanthemum is self-incompatible [1,5]. Therefore, understanding the mechanism of self-incompatibility (SI) is crucial for the development of chrysanthemum pure lines.

Self-incompatibility, also called self-infertility (SI), means the inability of a fully fertile hermaphroditic plant to produce zygotes when self-pollinated [6]. It is distributed widely [7] throughout all the principal lineages of angiosperms, being present in approximately 19 orders, 71 families and 250 genera comprising approximately 60% of all angiosperm species. Therefore, the study of SI is always the hotspot of reproductive developmental biology, and scientists have made major achievements in this field.

Most SI systems are controlled by a single highly polymorphic locus, the *S*-locus [8,9]. The *S*-locus contains a minimum of two tightly linked polymorphic genes, one controlling pollen identity and the other controlling pistil identity. In addition to these, many other genes are known or predicted to reside at the *S*-locus [10]. Genetic data also show that genes not linked to the *S*-locus are important for fully functional SI [11]. These genes encode proteins of diverse function, many of which are involved in signaling pathways downstream of the S-protein-mediated self-recognition machinery [12]. According to the genetic control of the incompatibility phenotype in pollen, SI systems are divided into two broad categories: gametophytic self-incompatibility (GSI) and sporophytic self-incompatibility (SSI). In GSI, incompatibility phenotype of the pollen is determined by the pollen’s own haploid genome. GSI systems are the most abundant SI system in flowering plants. There are two major GSI systems characterized at the molecular level, one is an S-RNase-based system of Solanaceae [13,14], Scrophulariaceae [15,16] and Rosaceae [17,18], and the other is a Ca^2+^-dependent system of Papaveraceae [19,20]. In SSI, incompatibility phenotype of the pollen is determined by the diploid genome of the parental plant. SSI is much less widespread than GSI, and it is unknown how many mechanistically different types of SSI systems are acting on flowering plants. Among these, Brassicaceae SSI [21,22] is the most studied system. Like Papaver GSI system, it is controlled by a ligand/receptor interaction, but the ligand (SCR/SP11—*S*-locus cysteine-rich protein/*S*-locus protein 11) is carried by the pollen grain and the receptor (SRK—*S*-locus receptor kinase) resides in the epidermal (papilla) cells of the stigma.

In our previous study, we have got a self-compatible chrysanthemum cultivar, “Q10-33-1”. In this study, in order to investigate cellular reasons for differences in fertility among progenies, four progenies of it with different seed set were used as materials. We carried out a systematical investigation on pollen morphology, pollen germination percentage, pistil receptivity and embryo development of them. After that, self-compatible and self-incompatible progenies could be separately picked out. For the purpose of finding out molecular reasons for self-incompatibility, the two progenies were used as materials. We applied RNA sequencing technique, and then analyzed their transcriptomic profiles to find out stigma and anther-expressed genes and their correlation.

## 2. Results

### 2.1. Flowering Traits of “Q10-33-1”’s Four Progenies

**Figure 1:**
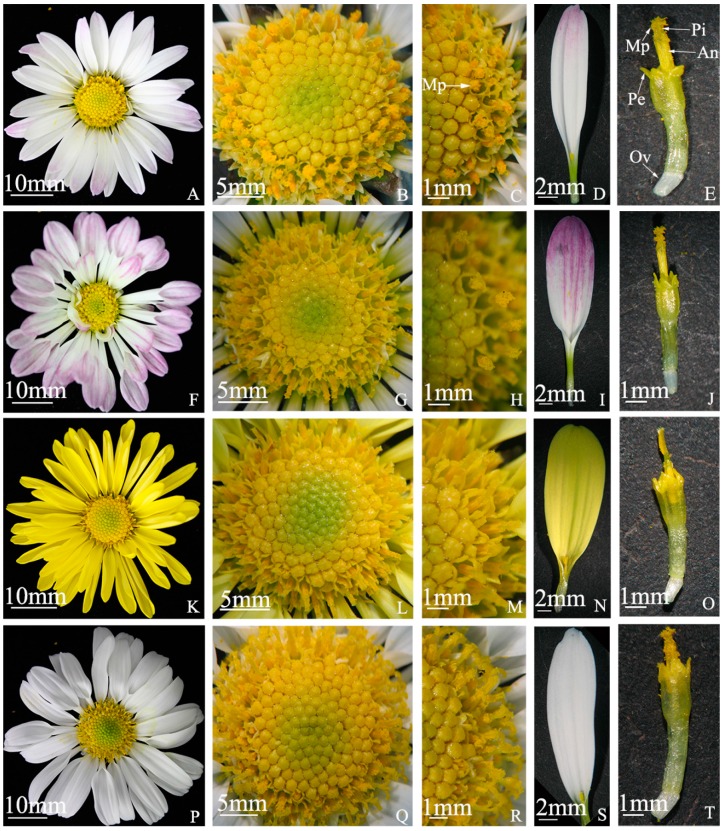
Flowering traits of “Q10-33-1”’s four progenies. (**A**–**E**) “Q10-33-1①”; (**F**–**J**) “Q10-33-1③”; (**K**–**O**) “Q10-33-1④”; (**P**–**T**) “Q10-33-1⑩”. The first column is the whole inflorescence, the second column is the whole tubular flower, the third column is a part of the tubular flower, the fourth column is the ray floret, and the last column is the tubiform floret. Abbreviations: Mp: mass of pollen grains, An: anther, Pi: pistil, Pe: petal, Ov: ovary.

There were small differences in flowering traits among “Q10-33-1”’s four progenies (Table 1). The ray florets of these progenies were almost white or yellow, and the inflorescence sizes were similar. The results indicated that they were really “Q10-33-1”’s progenies, and that it would be beneficial to analyze differences in self-fertility because of similar genetic background. Most worthy of mention is, pollen quantities of “Q10-33-1①”, ”Q10-33-1③” and “Q10-33-1⑩” were much more than that of “Q10-33-1⑩” (Figure 1). This showed that seed set of “Q10-33-1①”, ”Q10-33-1③” and “Q10-33-1④” might be connected with their pollen quantities, but seed set of “Q10-33-1⑩” was not connected with its pollen quantity.

### 2.2. Pollen Morphology and Pollen Germination Percentages of “Q10-33-1”’s Four Progenies

**Figure 2:**
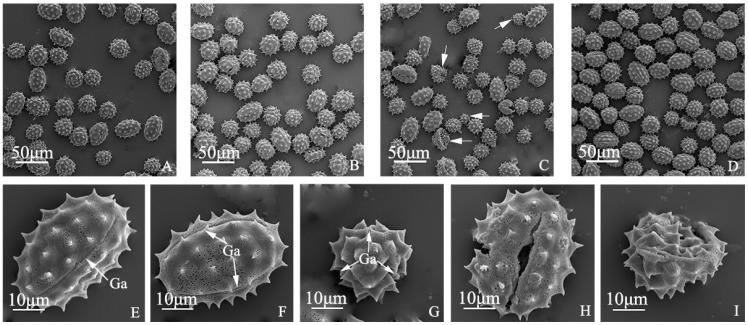
Pollen morphology of “Q10-33-1”’s four progenies. (**A**) “Q10-33-1①”; (**B**) “Q10-33-1③”; (**C**) “Q10-33-1④”, the white arrows indicated abnormal pollen grains; (**D**) “Q10-33-1⑩”; (**E**–**G**) three germinal apertures in equatorial and polar view; (**H**, **I**) Abnormal pollen morphology. Abbreviations: Ga: germinal aperture.

**Figure 3:**
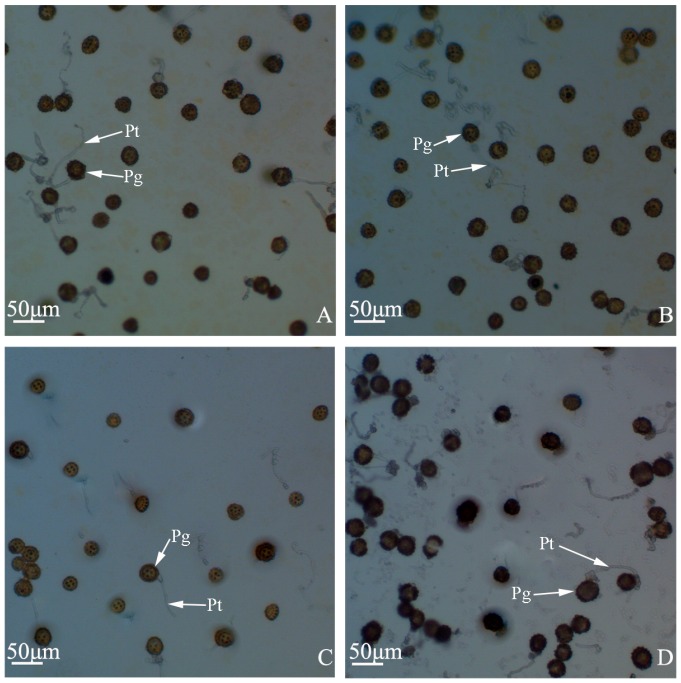
Pollen germination percentages of “Q10-33-1”’s four progenies. (**A**) “Q10-33-1①”; (**B**) “Q10-33-1③”; (**C**) “Q10-33-1④”; (**D**) “Q10-33-1⑩”. Abbreviations: Pg: pollen grain, Pt: pollen tube.

The pollen grains of “Q10-33-1”’s four progenies were prolate, elliptic in the equatorial view, and trifid round in the polar view. They had three germinal apertures, and the surface was coated with spinous glyphs and meshes (Figure 2E–G). There were abnormal pollen grains (Figure 2H,I) in all four progenies. Among these, the number of abnormal pollen grains in “Q10-33-1⑩” was the least, while the number of abnormal pollen grains in “Q10-33-1④” was the most (Figure 2A–D). Meanwhile, pollen germination percentage of ‘Q10-33-1⑩’ was the highest, the second was “Q10-33-1①”, the next was “Q10-33-1③”, and pollen germination percentage of “Q10-33-1④” was the lowest (Table 2, Figure 3). This supported the view that pollen morphology directly affected pollen germination percentage. Simultaneously, the results indicated that except for “Q10-33-1⑩”, seed set of “Q10-33-1①”, “Q10-33-1③” and “Q10-33-1④” were connected with their pollen germination percentages.

### 2.3. Pistil Receptivity of “Q10-33-1”’s Four Progenies

**Figure 4:**
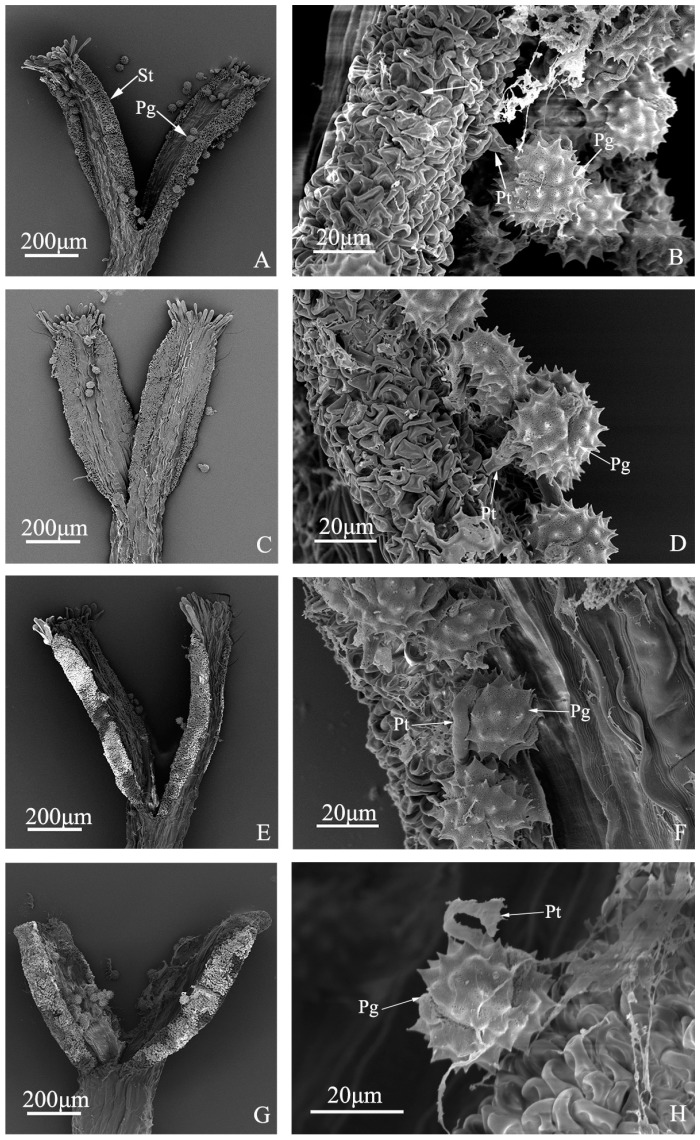
Pollen tube growth of “Q10-33-1”’s four progenies at ultrastructural level. (**A**, **B**) “Q10-33-1①”; (**C**, **D**) “Q10-33-1③”; (**E**, **F**) “Q10-33-1④”; (**G**, **H**) “Q10-33-1⑩”; (**B**, **D**, **F**) Pollen tube grew into the stigma; (**H**) Pollen tube stopped growing and failed to penetrate the stigma’s surface. Abbreviations: St: stigma, Pg: pollen grain, Pt: pollen tube.

In previous study, we found that average numbers of pollen grains germinating on each stigma among “Q10-33-1”’s ten progenies were nearly positively proportional to their seed set. Among these, the average numbers of pollen grains germinating on each stigma of “Q10-33-1①”, “Q10-33-1③”, “Q10-33-1④” and “Q10-33-1⑩” were 30.43, 8.57, 5.33 and 7.20, respectively. Thus it could be seen, except for “Q10-33-1⑩,” the average numbers of pollen grains germinating on each stigma among other three progenies were positively proportional to their seed set. In this study, the pistil receptivity of “Q10-33-1”s four progenies was observed at the ultrastructural level (Figure 4). It was found that pollen tube stopped growing and failed to penetrate the stigma’s surface in “Q10-33-1⑩” (Figure 4H). This indicated that abnormal pistil receptivity was the main reason leading to the failure of seed set in “Q10-33-1⑩.”

### 2.4. Ovary Development of “Q10-33-1”’s Four Progenies

In all four progenies, the percentage of full ovaries were nearly negatively proportional to the days after self-pollination (Table 3), indicating that ovary abortion occurred gradually with the extension of the days after self-pollination. Meanwhile, the percentage of full ovaries among four progenies were nearly positively proportional to their seed set (Table 3), showing that ovary development of “Q10-33-1”’s four progenies was directly related to their seed set. Among the four progenies, no full ovary was found in “Q10-33-1⑩,” and pollen tube growth of this progeny was abnormal, these might come to a conclusion that “Q10-33-1⑩” was self-incompatible.

**Figure 5:**
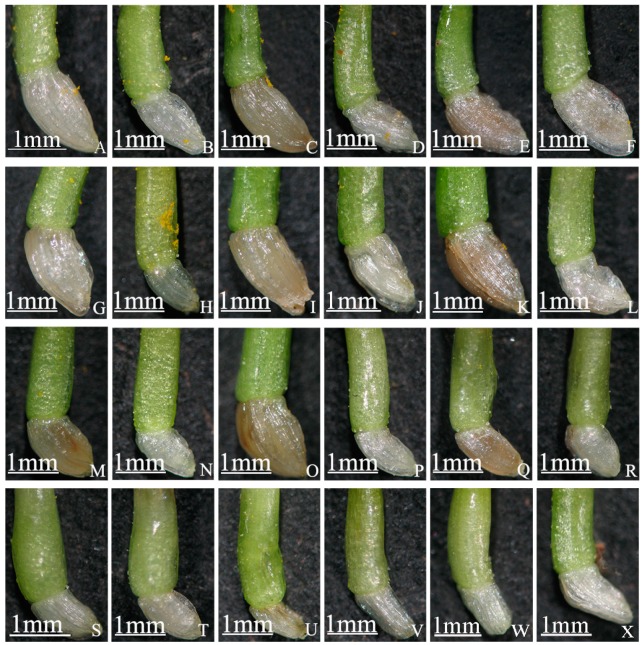
Full and unful ovaries at different days after self-pollination in “Q10-33-1”’s four progenies. (**A**–**F**) “Q10-33-1①”; (**G**–**L**) “Q10-33-1③”; (**M**–**R**) “Q10-33-1④”; (**S**–**U**) “Q10-33-1⑩”; (**V**–**X**) Ovaries without pollination; (**A**, **G**, **M**) Full ovaries at 12 days after self-pollination; (**B**, **H**, **N**, **S**) Unful ovaries at 12 days after self-pollination; (**C**, **I**, **O**) Full ovaries at 18 days after self-pollination; (**D**, **J**, **P**, **T**) Unful ovaries at 18 days after self-pollination; (**E**, **K**, **Q**) Full ovaries at 30 days after self-pollination; (**F**, **L**, **R**, **U**) Unful ovaries at 30 days after self-pollination.

In addition, the differences between full and unful ovaries on different days after self-pollination were observed under a stereoscope. The ovule of a full ovary was also full and gradually became dark in color until seed formation, while the ovule of an unful ovary was smaller, the color was white, and it had no tendency to form seed (Figure 5).

### 2.5. Embryo Development of “Q10-33-1”’s Three Progenies at Microscopic Level

**Figure 6:**
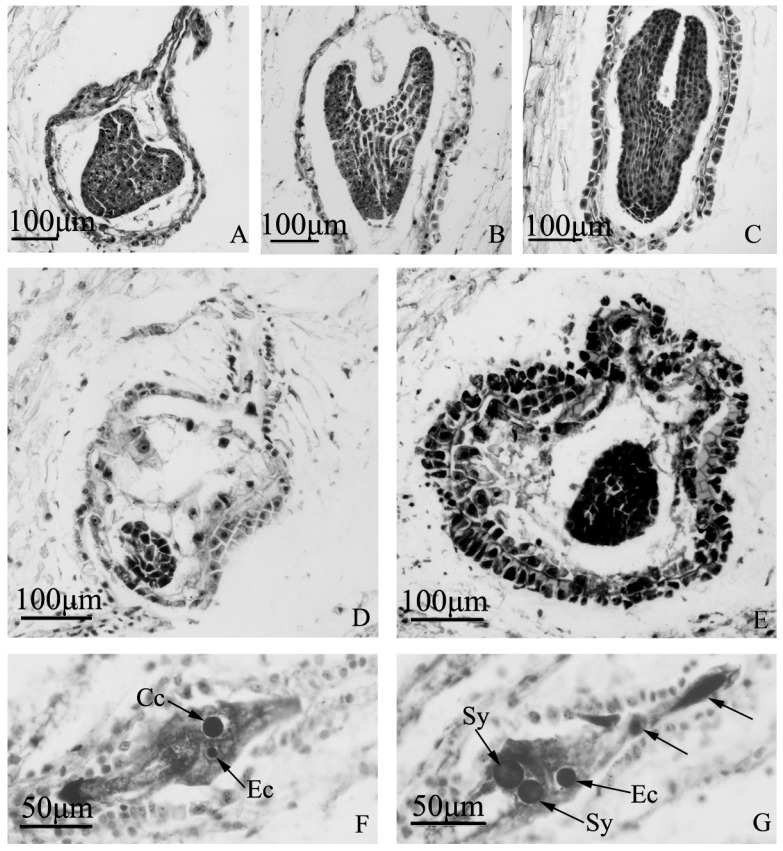
Embryo development of “Q10-33-1”’s three progenies at microscopic level. (**A**–**C**) Normal embryo development of “Q10-33-1①”; (**A**) Heart embryo at 12 days after self-pollination; (**B**) Torpedo embryo at 18 days after self-pollination; (**C**) Cotyledon embryo at 18 days after self-pollination; (**D**, **E**) Embryo abortion of “Q10-33-1④”; (**D**) Embryo abortion at 12 days after self-pollination, the embryo was degenerating; (**E**) Embryo abortion at 18 days after self-pollination, the embryo was degenerating and the integument was thickening; (**F**, **G**) Unfertilized ovules of “Q10-33-1⑩”, tetranucleate embryo sacs, two black arrows indicated two degenerated megaspores. Abbreviations: Cc: central cell, Ec: egg cell, Sy: synergid.

If the process of embryo development was normal, heart embryos could be seen in ovaries at 12 days after self-pollination, and torpedo or cotyledon embryos could be seen in ovaries at 18 days after self-pollination (Figure 6A–C). However, embryo abortion occurred in some ovaries with the extension of the days after self-pollination (Figure 6D,E). Meanwhile, no embryos could be seen in “Q10-33-1⑩”, we could only find tetranucleate embryo sacs, showing one central cell, one egg cell, two synergids near the micropylar end and two degenerated megaspores (Figure 6F,G).

### 2.6. Embryo Development of “Q10-33-1①” at Ultrastructural Level

**Figure 7:**
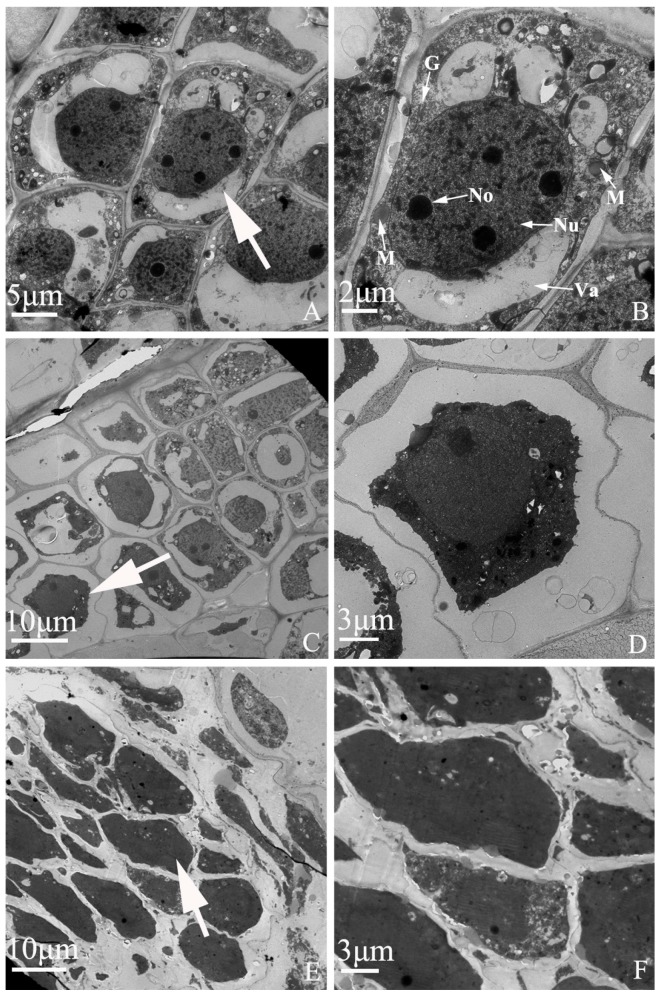
Embryo development of “Q10-33-1①” at ultrastructural level. (**A**, **B**) Normal embryonic cells; (**C**, **D**) Abortive embryonic cells at 12 days after self-pollination; (**E**, **F**) Abortive embryonic cells at 18 days after self-pollination. (**B**, **D**, **F**) were partial enlarged views of cells pointed by the white arrows in (**A**, **C**, **E**). Abbreviations: Nu: nucleus, No: nucleolus, M: mitochondria, G: Golgi complex, Va: vacuole.

The results of TEM showed that there were significant differences between normal and abortive embryonic cells (Figure 7). In normal embryonic cells, the nucleus and nucleolus could be seen clearly and some typical cell organelles such as mitochondria, Golgi complex and vacuoles were also found (Figure 7A,B). Meanwhile, at the early stage of embryo abortion, cytoplasmic vacoulation, condensed cytoplasm and degradative organelles were observed in abortive embryonic cells (Figure 7C,D). At late stage of embryo abortion, the nucleus and organelles were fully degraded. No clear cellular tissues could be seen in abortive embryonic cells (Figure 7E,F).

### 2.7. Transcriptome Sequencing and Read Assembly

To study transcriptomic gene expression differences between mature stigmas of self-compatible and self-incompatible progenies, two cDNA libraries from 3S (mature stigmas of “Q10-33-1③”) and 10S (mature stigmas of “Q10-33-1⑩”) were subjected to Illumina sequencing. After reads filtering, we obtained 44.52 Mb and 44.19 Mb clean reads from two cDNA libraries, containing 6.68 Gb and 6.63 Gb clean bases, respectively. The clean reads ratios of the two samples were about 80%, the Q20 percentages were more than 97% and the Q30 percentages were more than 91% (Table 4). After clustering the high-quality reads, we finally obtained 113,800 unigenes with a total length of 94,376,715 bp, a mean length of 829 bp and a GC percentage of 39.40% (Table 5).

To study transcriptomic gene expression differences between mature anthers of self-compatible and self-incompatible progenies, two cDNA libraries from 3A (mature anthers of “Q10-33-1③”) and 10A (mature anthers of “Q10-33-1⑩”) were subjected to Illumina sequencing. After reads filtering, we obtained 44.25 Mb and 44.37 Mb clean reads from two cDNA libraries, containing 6.64 Gb and 6.65 Gb clean bases, respectively. The clean reads ratios of the two samples were about 80%, the Q20 percentages were more than 97% and the Q30 percentages were more than 91% (Table 6). After clustering the high-quality reads, we finally obtained 113,638 unigenes with a total length of 91,284,692 bp, a mean length of 803 bp and a GC percentage of 39.66% (Table 7).

### 2.8. Unigene Functional Annotation

To predict the potential functions of unigenes in chrysanthemum stigmas, all assembled unigenes were subjected to functional annotation using 7 functional databases, including NR, NT, Swiss-Prot, KEGG, COG, Interpro and GO. Consequently, 61,731 unigenes were annotated with the databases, and the number of unigenes annotated with each database was 57,127, 42,115, 39,577, 42,586, 19,476, 38,563 and 23,423, respectively (Table 8).

**Figure 8:**
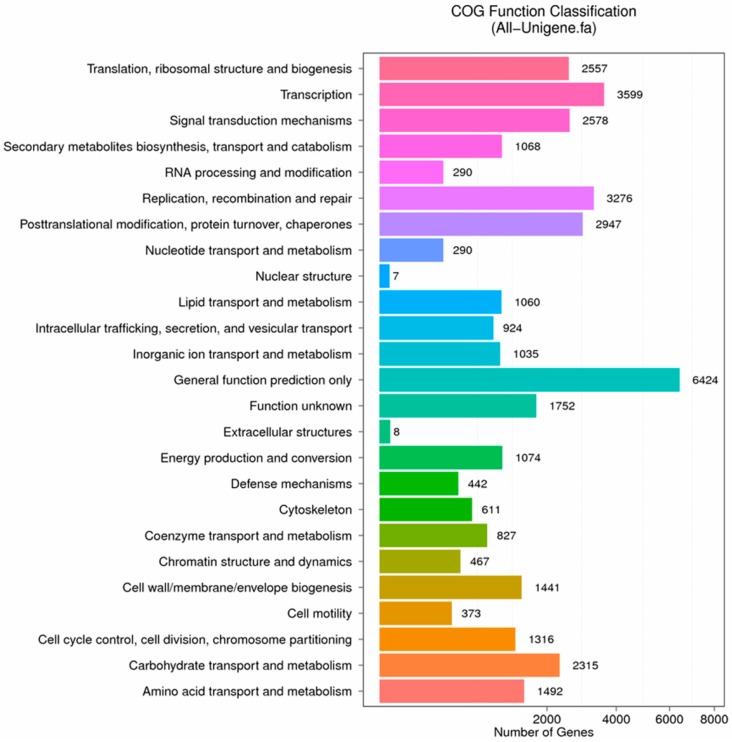
COG function classification of stigma transcriptome. *X* axis represents the number of Unigenes. *Y* axis represents the COG functional category.

In order to understand the functions of unigenes intuitively and effectively, 19,476 unigenes, accounting for 17.11% of the total annotated unigenes, were further annotated and classified with COG database. Among the 25 COG categories, the most abundant was “general function prediction only” (6424, 32.98%), followed by “transcription” (3599, 18.48%), “replication, recombination and repair” (3276, 16.82%) and “posttranslational modification, protein turnover, chaperones” (2947, 15.13%). The least two were “nuclear structure” (7, 0.036%) and “extracellular structures” (8, 0.041%) (Figure 8).

**Figure 9:**
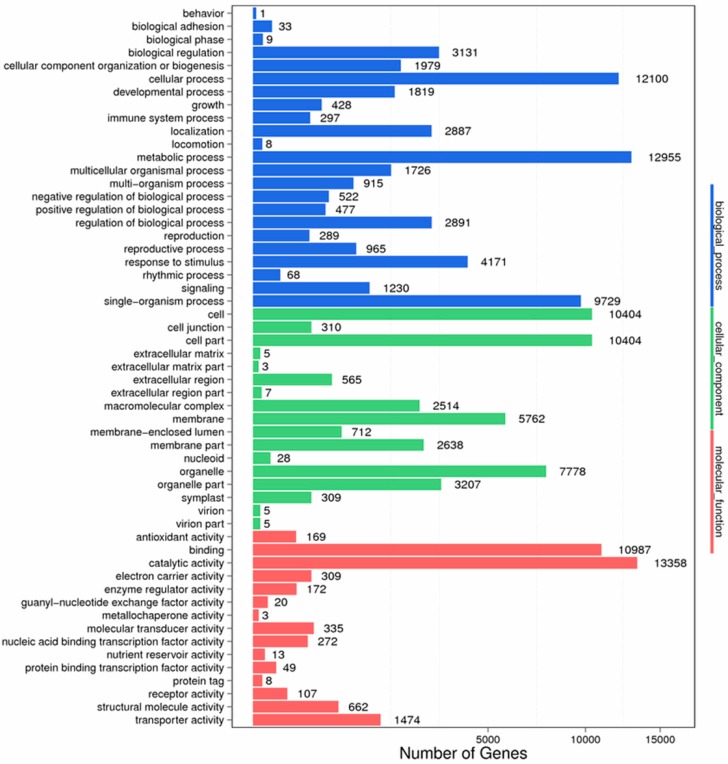
GO function classification of stigma transcriptome. *X* axis represents the number of Unigenes. *Y* axis represents the GO functional category.

According to the NR annotation, we used GO assignments to classify the functions of unigenes. As a result, 23,423 unigenes were classified into 55 functional subcategories, which belonged to 3 main categories: biological process, cellular component and molecular function. There were 23, 17 and 15 functional groups in each category, respectively (Figure 9). In the biological process category, “metabolic process” and “cellular process” were the main functional subcategories. In terms of cellular component category, two major subcategories were “cell” and “cell part”. For the molecular function category, “catalytic activity” and “binding” were remarkable.

Finally, KEGG annotation was performed to identify the biological pathways activated in chrysanthemum’s mature stigmas. A total of 42,586 unigenes were assigned to 135 KEGG pathways (Table S3). The majority of these pathways were “metabolic pathways (ko01100)” (21.41%), “biosynthesis of secondary metabolites (ko01110)” (12.27%), “plant-pathogen interaction (ko04626)” (4.02%) and “endocytosis (ko04144)” (3.69%).

To predict potential functions of unigenes in chrysanthemum anthers, all assembled unigenes were performed functional annotation with 7 functional databases, including NR, NT, Swiss-Prot, KEGG, COG, Interpro and GO. Consequently, 63,517 unigenes were annotated with the databases, and the number of unigenes annotated with each database was 59,346, 42,785, 40,774, 43,970, 20,580, 40,653 and 24,313, respectively (Table 9).

**Figure 10:**
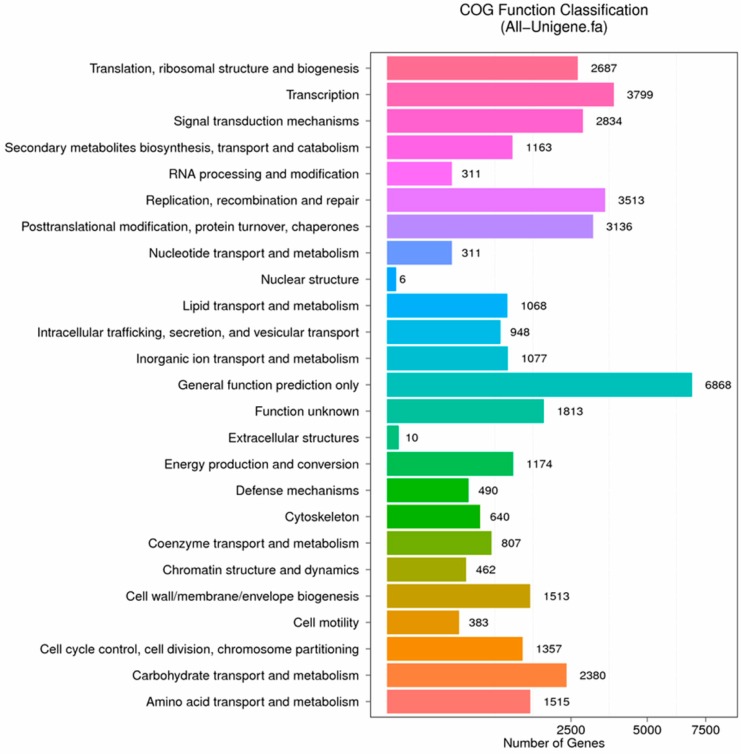
COG function classification of anther transcriptome. *X* axis represents the number of Unigenes. *Y* axis represents the COG functional category.

In order to understand the functions of unigenes intuitively and effectively, 20,580 unigenes, accounting for 18.11% of the total annotated unigenes, were further annotated and classified with COG database. Among the 25 COG categories, the most abundant was “general function prediction only” (6868, 33.37%), followed by “transcription” (3799, 18.46%), “replication, recombination and repair” (3513, 17.07%) and “posttranslational modification, protein turnover, chaperones” (3136, 15.24%). The least two were “nuclear structure” (6, 0.029%) and “extracellular structures” (10, 0.049%) (Figure 10).

**Figure 11:**
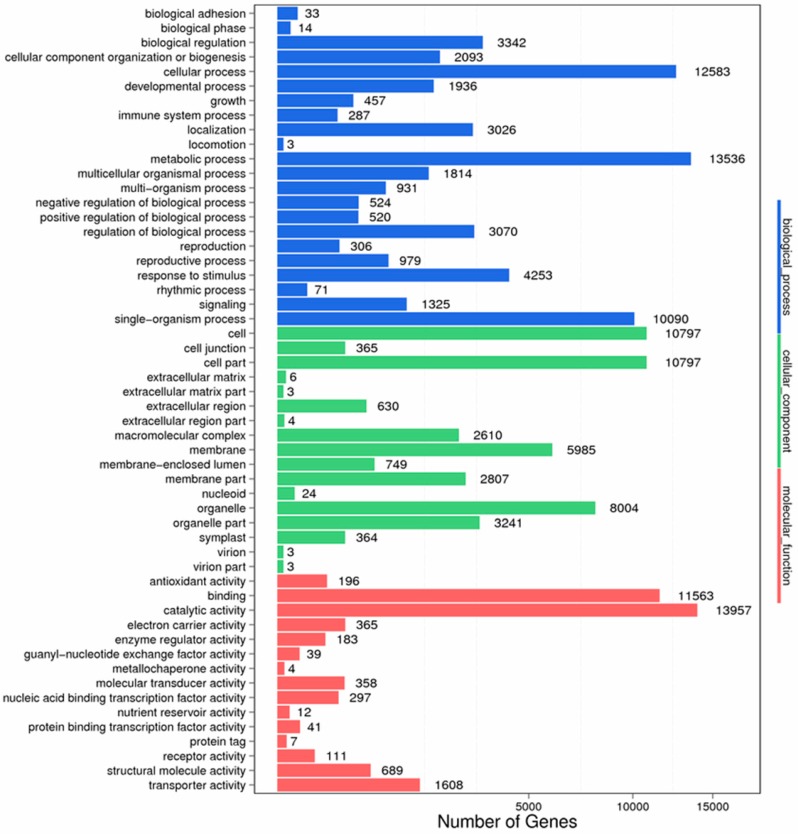
GO function classification of anther transcriptome. *X* axis represents the number of Unigenes. *Y* axis represents the GO functional category.

According to the NR annotation, we used GO assignments to classify the functions of unigenes. As a result, 24,313 unigenes were classified into 54 functional subcategories, which belonged to 3 main categories: biological process, cellular component and molecular function. There were 22, 17 and 15 functional groups in each category, respectively (Figure 11). In the biological process category, “metabolic process” and “cellular process” were the main functional subcategories. In terms of cellular component category, two major subcategories were “cell” and “cell part”. For the molecular function category, “catalytic activity” and “binding” were remarkable.

Finally, KEGG annotation was performed to identify the biological pathways activated in chrysanthemum’s mature anthers. A total of 43,970 unigenes were assigned to 135 KEGG pathways (Table S4). The majority of these pathways were “metabolic pathways (ko01100)” (21.36%), “biosynthesis of secondary metabolites (ko01110)” (12.16%), “endocytosis (ko04144)” (4.14%) and “plant-pathogen interaction (ko04626)” (3.95%).

### 2.9. DEGs (Differently Expressed Genes) Related to SI in Mature Stigmas and Anthers of Self-Compatible and Self-Incompatible Progenies

Based on the FPKM (fragments per kb per million fragments) value, we explored gene expression level in 3S and 10S. Compared with 3S, in 10S, 8130 and 3766 genes were up- and down-regulated, respectively. The details about these DEGs were shown in Table S5. To determine putative functions of the DEGs, GO and KEGG analysis were applied. As a consequence, 7483 DEGs were associated with GO categories, 7194 DEGs were mapped to 134 KEGG pathways (Table S6). The high number of DEGs provided an abundant list of candidate SI-related genes in stigmas.

Based on the FPKM value, we explored gene expression level in 3A and 10A. Compared with 3A, in 10A, 7287 and 4538 genes were up- and down-regulated, respectively. The details about these DEGs were shown in Table S7. To determine putative functions of the DEGs, GO and KEGG analysis were applied. As a consequence, 7657 DEGs were associated with GO categories, 7020 DEGs were mapped to 133 KEGG pathways (Table S8). The high number of DEGs provided an abundant list of candidate SI-related genes in anthers.

After deep analysis and screening of these DEGs, some potential stigma *S* genes and pollen *S* genes were found out, they were listed in Table 10 and Table 11, respectively.

### 2.10. Validation of Gene Expression Profiles by qRT-PCR

**Figure 12:**
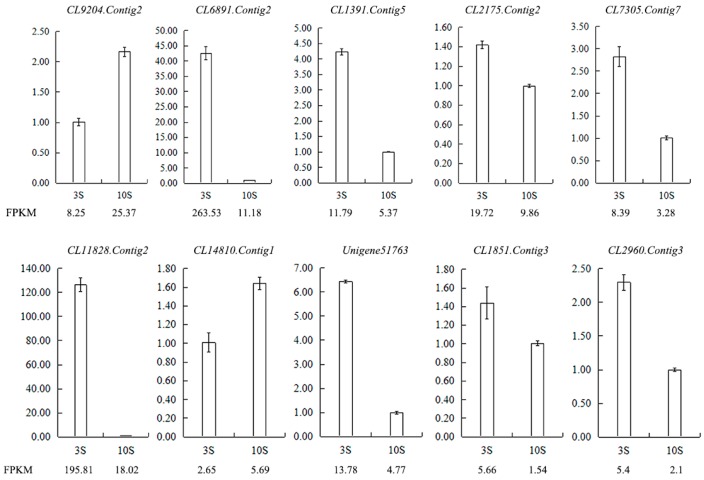
Validation of the RNA-seq results for stigma transcriptome libraries by qRT-PCR. FPKM is their abundance in the sequencing data of the transcriptome libraries. Abbreviations: CL9204.Contig2: ribonuclease 1, ribonuclease T2 family, CL6891.Contig2: epidermis-specific secreted glycoprotein EP1, CL1391.Contig5: G-type lectin *S*-receptor-like serine/threonine-protein kinase SD1-1, CL2175.Contig2: *S*-receptor kinase-like protein 1, CL7305.Contig7: G-type lectin *S*-receptor-like serine/threonine-protein kinase SD1-29, CL11828.Contig2: G-type lectin *S*-receptor-like serine/threonine-protein kinase RLK1, CL14810.Contig1: G-type lectin *S*-receptor-like serine/threonine-protein kinase At1g67520, Unigene51763: *S*-receptor kinase-like protein 1, CL1851.Contig3: G-type lectin *S*-receptor-like serine/threonine-protein kinase SD1-1, CL2960.Contig3: G-type lectin *S*-receptor-like serine/threonine-protein kinase At4g27290.

In the library of stigmas, ten differentially expressed genes were selected for qRT-PCR to test the reliability of RNA-seq data. The result showed that the expression trend of almost all genes was consistent with sequencing data (Figure 12). Most of DEGs selected were related to SI female determinants in other plant families, such as *S*-receptor-like serine/threonine-protein kinase, protein in ribonuclease T2 family and epidermis-specific secreted glycoprotein.

**Figure 13:**
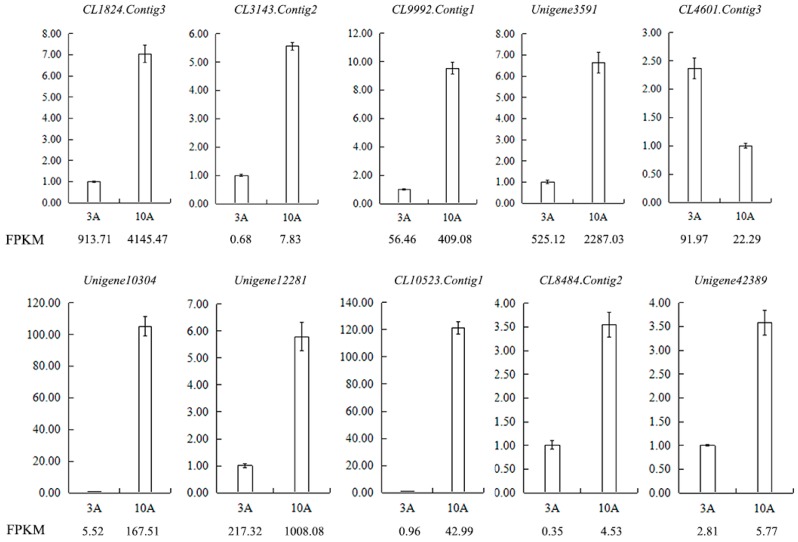
Validation of the RNA-seq results for anther transcriptome libraries by qRT-PCR. FPKM is their abundance in the sequencing data of the transcriptome libraries. Abbreviations: CL1824.Contig3: pollen-specific protein SF3, CL3143.Contig2: participates in pollen germination and pollen tube growth, CL9992.Contig1: regulates pollen tube growth, Unigene3591: participates in pollen tube growth, CL4601.Contig3: pollen-specific leucine-rich repeat extensin-like protein 1, Unigene10304: *S* locus-related glycoprotein 1 binding pollen coat protein (SLR1-BP), Unigene12281: pollen coat-like protein, CL10523.Contig1: *S* locus-related glycoprotein 1 binding pollen coat protein (SLR1-BP), CL8484.Contig2: SKP1-like protein 1A, Unigene42389: Cullin-1.

In the library of anthers, ten differentially expressed genes were also selected for qRT-PCR to test the reliability of RNA-seq data. The result showed that the expression trend of almost all genes was consistent with sequencing data (Figure 13). Most of DEGs selected were related to pollen germination, pollen tube reception and growth, and SI male determinants in other plant families, such as pollen coat protein.

## 3. Discussion

### 3.1. Cellular Mechanism of Differences in Fertility among Progenies

This study shows that the self-pollinated seed set of chrysanthemum is mainly affected by pollen germination percentage, the germination behavior of pollen grains on stigmas, pollen tube growth and embryo development.

Pollen germination percentage has some effects on seed set [24,25], while abnormal pollen morphology can cause low pollen germination percentage. In “Q10-33-1”s four progenies, the number of abnormal pollen grains in “Q10-33-1④” was the largest, so its pollen germination percentage was the lowest; while the number of abnormal pollen grains in “Q10-33-1⑩” was the least, so its pollen germination percentage was the highest. In addition, except for “Q10-33-1⑩”, pollen germination percentages of other three progenies were positively proportional to their seed set. This indicated that pollen germination percentage indeed had an effect on seed set. However, seed set of “Q10-33-1⑩” was not connected with its pollen germination percentage. Its pollen germination percentage was the highest in four progenies, but its seed set was 0. This indicated that “Q10-33-1⑩”s pollen germination percentage was not the main reason for its self-pollinated failure.

In previous study, we found that the interaction between some chemical products secreting from stigmas and those secreting from pollen walls was incompatible, and consequently prevented pollen grains from germinating normally on stigmas, resulting in low seed set [26]. Therefore, the germination behavior of pollen grains on stigmas also has an effect on seed set [27,28,29]. In “Q10-33-1”s four progenies, except for “Q10-33-1⑩”, the average numbers of pollen grains germinating on each stigma among other three progenies were positively proportional to their seed set. This result just confirmed the above view.

Moreover, pollen tube growth also affects seed set [6,30,31]. If pollen tube can reach ovary through style to finish double fertilization, an embryo will be formed, otherwise, no embryo will be formed. In “Q10-33-1①” and “Q10-33-1③” and “Q10-33-1④”, most pollen tube growth was normal, while in “Q10-33-1⑩”, most pollen tube growth was abnormal. Thus, we came to the conclusion that abnormal pollen tube growth was the key reason for self-pollinated failure of “Q10-33-1⑩”.

Lastly, embryo development is another important factor related to seed set [32,33,34]. Except for “Q10-33-1⑩”, embryo abortion was observed in other three progenies, this indicated that embryo development also really had an effect on seed set.

In conclusion, seed set of: “Q10-33-1①”, “Q10-33-1③", and “Q10-33-1④” were related to pollen germination percentage, the germination behavior of pollen grains on stigmas and embryo development. Meanwhile, seed set of “Q10-33-1⑩” was mainly affected by pollen tube growth. We also came to a conclusion that “Q10-33-1⑩” was self-incompatibility. In addition, as shown in the Figure 4H, the inhibition of pollen tube growth in “Q10-33-1⑩” was taken place on the surface of stigma, rather than in the style. This indicated that SI in chrysanthemum was more likely to SSI.

### 3.2. Molecular Mechanism of SI

In molecular study, SI of angiosperm is mainly controlled by allele-specific interactions between two highly polymorphic *S*-determinants. So far, however, little research has been done on chrysanthemum SI. In our transcriptome data, some DEGs related to *S*-determinants in other plant families were found, and they might also play an important role in chrysanthemum SI.

According to a previous study, GSI was found typically in Solanaceae, Rosaceae, Scrophulariaceae and Papaveraceae, whereas SSI was found in Brassicaceae, Asteraceae and Convolvulaceae [8]. The most studied SSI system is Brassicaceae SSI. In this family, the female *S*-determinant is SRK (*S*-receptor serine/threonine kinase), moreover, SLG (*S*-locus glycoprotein) also plays a supporting role in SI. The male *S*-determinant is SCR (*S*-locus cysteine-rich protein)/SP11 (*S*-locus protein 11), which is a kind of pollen coat protein [35]. This may provide a key clue for analyzing SI of chrysanthemum. In the analysis of stigma transcriptome, some unigenes encoding *S*-receptor-like serine/threonine-protein kinase or *S*-locus glycoprotein were up-regulated in 10S, such as *Unigene8775* (4.3 in 10S, 0.84 in 3S), *Unigene12923* (8.86 in 10S, 3.51 in 3S) and so on. Meanwhile, in the analysis of anther transcriptome, some unigenes encoding pollen coat-like protein were up-regulated in 10A, such as *CL10523.Contig1* (42.99 in 10A, 0.96 in 3A), *Unigene12281* (1008.08 in 10A, 217.32 in 3A) and so on. That is, the unigenes related to *S*-determinants in Brassicaceae were up-regulated in self-incompatible chrysanthemum progeny. The results raise a hypothesis that SI system of chrysanthemum is similar to Brassicaceae SSI system.

Although it was reported that SSI was found in Asteraceae, there was no precise evidence to confirm it. Maybe GSI system of Rosaceae also plays an important role in chrysanthemum’s SI. In this GSI system, the female *S*-determinant is S-RNase (*S* ribonuclease), belonging to ribonuclease T2 family, and the male *S*-determinant is SFB/SLF (*S*-locus F-box protein) [36,37,38]. In the comparative analysis of stigma transcriptome, some unigenes encoding ribonuclease belonging to ribonuclease T2 family were up-regulated in 10S, such as *CL9204.Contig2* (25.37 in 10S, 8.25 in 3S), *Unigene9014* (120.17 in 10S, 65.25 in 3S) and so on. Simultaneously, in the analysis of anther transcriptome, some unigenes encoding F-box protein were up-regulated in 10A, such as *CL442.Contig8* (0.73 in 10A, 0 in 3A), *CL442.Contig9* (1.87 in 10A, 1.1 in 3A) and so on. In addition, in anther transcriptome analysis, some unigenes encoding Skp1 (S-phase kinase-associated protein 1)-like protein and Cullin1-like protein were up-regulated in 10A, such as *CL8484.Contig2* (4.53 in 10A, 0.35 in 3A) and *Unigene42389* (5.77 in 10A, 2.81 in 3A). The three kinds of proteins, F-box, Skp1 and Cullin1, can form a SCF complex (Skp, Cullin, F-box containing complex), this complex plays a pivotal role in S-RNase-based SI system [36]. Likewise, these results raise a hypothesis, that is, S-RNase-based GSI system may also function in chrysanthemum SI.

Furthermore, in stigma transcriptome analysis, some unigenes participating in recognition of pollen were found, such as *CL1851.Contig3* and *CL2175.Contig6*. We also found some unigenes encoding specific proteins which had been reported in stigma transcriptome files of other plants [39,40]. For instance, *CL9691.Contig3* and *Unigene23027* encoded stigma-specific peroxidase, *CL831.Contig1* and *Unigene47365* encoded mitogen-activated protein kinase (MAPK), and *CL916.Contig6* and *Unigene590* encoded pistil-specific extensin-like protein. Meanwhile, on the basis of previous studies [41,42], in another transcriptome analysis, some unigenes participating in pollen germination, pollen exine formation, pollen tube reception and pollen tube growth were found. For example, *CL500.Contig1* and *CL3143.Contig2* participated in pollen germination, *CL12767.Contig2* took part in pollen exine formation, *CL6315.Contig9* participated in pollen tube reception, and *CL9992.Contig1* took part in pollen tube growth. The above unigenes may also play an important role in chrysanthemum SI.

From the above, we surmise that in chrysanthemum SI, the SSI system—similar to Brassiceae SI—plays a main role, and S-RNase-based SI system may play a supporting role.

## 4. Materials and Methods

### 4.1. Experimental Materials

Four progenies of “Q10-33-1” with different seed set were used as materials for cellular experiments: “Q10-33-1①” (the seed set was 37.23%), “Q10-33-1③” (the seed set was 26.77%), “Q10-33-1④” (the seed set was 7.97%), and “Q10-33-1⑩” (the seed set was 0). Among them, two progenies were used as materials for molecular experiments: “Q10-33-1③” (self-compatible) and “Q10-33-1⑩” (self-incompatible). All materials were grown in Chrysanthemum Germplasm Resource Preserving Center, Nanjing Agricultural University, Nanjing, China (32°05′ N, 118°90′ E).

### 4.2. Determination of Flowering Traits

On the basis of relative studies of other researchers [43,44,45], we selected some well-grown inflorescences and measured their flowering traits, including flower color, petal shape, anthotaxy diameter, flower disc diameter, length and width of ray floret, and the number of ray florets and tubiform florets. Each trait was determined five times. In addition, we observed and photographed the inflorescence, tubiform floret and ray floret of it under a SLR (single lens reflex) camera and a stereoscope.

### 4.3. Ultrastructural Observation of Pollen Morphology

Abnormal pollen morphology may lead to low pollen germination percentage, thus affecting seed set. In order to support this view, fresh pollen grains were collected between 10:00 and 14:00 on a sunny day. The pollen grains were dried at 42 °C for 10–15 days, until they were not together [46,47,48]. Then the pollen grains were coated with gold, and observed under a scanning electron microscope.

### 4.4. Determination of Pollen Germination Percentage

We adopted in vitro culture method [26,49] to determine pollen germination percentage. A few drops of culture medium ME3 (0.1 g·L^−1^ H_3_BO_3_, 0.3 g·L^−1^ CaCl_2_·2H_2_O, 0.001 g·L^−1^ CoCl_2_·6H_2_O) + 200 g·L^−1^ PEG4000 were added on a glass slide, then fresh pollen grains were distributed on the glass slide. After that, the glass slide was incubated at 20 °C for about two hours, because it was found from the past research that pollen grains would not germinate after incubating two hours. Germinated pollen grains were observed under an Olympus BX41 microscope (Olympus Corporation, Tokyo, Japan) and germination percentage was counted from ten optical fields. When pollen tube length was longer than the diameter of the pollen grain, the pollen grain was identified as germinating. Each experiment was conducted three times. Notably, pollen germination percentage must be determined immediately after pollen grains collection, because as time goes on, the germination percentage will reduce rapidly.

### 4.5. Ultrastructural Observation of Pistil Receptivity

Five self-pollinated inflorescences were cut off from each progeny at 24 h after pollination, immediately fixed in 2.5% glutaraldehyde solution, and put under vacuum until submerged. Then they were stored at 4 °C until use. The pistils were dissected from the ray florets, dehydrated in an ethanol series, subjected to critical-point drying, and coated with gold. Samples were observed with a scanning electron microscope.

### 4.6. Examination of Embryo Development

Five inflorescences at 12, 18 and 30 days after self-pollination were respectively cut off from each progeny. Then full and unful ovaries of these inflorescences were separately counted and photographed under a stereoscope. Each inflorescence was a repetition. Meanwhile, about five inflorescences of “Q10-33-1①”, “Q10-33-1④” and “Q10-33-1⑩”—at 12 and 18 days after self-pollination—were respectively collected, immediately fixed in FAA solution (95% ethanol:distilled water: formalin:glacial acetic acid = 63:27:5:5), put under vacuum until submerged and then stored at 4 °C until use. Full and unful ovaries were separately dissected from florets, dehydrated in an ethanol series, and then embedded in paraffin wax. Sections were cut to a thickness of 8–10 μm and stained with Heidenhain’s hematoxylin (Heidenhain, Berlin, Germany) [50,51]. The sections then were observed and photographed under an Olympus BX41 microscope. In addition, five inflorescences of “Q10-33-1①” at 12 and 18 days after self-pollination were collected, fixed in 2.5% glutaraldehyde solution, put under vacuum until submerged, and stored at 4 °C until use. Full and unful ovules were separately dissected from ovaries, dehydrated in an ethanol series, subjected to critical-point drying, and coated with gold. Samples were examined with a transmission electron microscope, and all images were processed digitally.

### 4.7. Samples Collection and RNA Extraction

According to previous studies [52], expression of *S*-alleles was tissue specific and developmentally controlled. Therefore, in this study, mature stigmas (at the optimal pollination period) of “Q10-33-1③” and “Q10-33-1⑩” were separately collected as intercomparable samples for RNA extraction, meanwhile, mature anthers (at the period closest to pollinating period) of the two progenies were separately collected as another intercomparable samples for RNA extraction. After collection, the four samples, mature stigmas of “Q10-33-1③” (3S), mature stigmas of “Q10-33-1⑩” (10S), mature anthers of “Q10-33-1③” (3A) and mature anthers of “Q10-33-1⑩” (10A), were immediately frozen in liquid nitrogen and stored at −80 °C.

Total RNA was extracted using the Trizol reagent according to the manufacturer’s protocol (Takara Bio Inc., Otsu, Japan) [53]. The quantity and quality of total RNA were confirmed by Agilent 2100 RNA 6000 Kit (Agilent Technologies Inc., Santa Clara, CA, USA) and electrophoresis on 1% agarose gel [53,54].

### 4.8. cDNA Library Construction and Illumina Sequencing

Illumina sequencing was performed at Beijing Genomics Institute (BGI)-Shenzhen, China according to the manufacturer’s instructions (Illumina, San Diego, CA, USA). Each cDNA sequencing library was constructed with the RNA mixture from three biological replicates.

### 4.9. Sequencing Reads Filtering and De Novo Assembly

The sequencing reads which containing low-quality, adaptor-polluted and high content of unknown base reads, should be processed to remove these reads before downstream analysis [55].

After filtering, we obtained clean reads from raw data, then Trinity [56] was applied to perform de novo assembly with clean reads. Next, Tgicl [57] was used to cluster transcripts to unigenes. The transcriptome datasets are available in the NCBI repository, (https://submit.ncbi.nlm.nih.gov/subs/sra/?from) Accession No. for library SRP131835.

### 4.10. Unigene Functional Annotation

Unigene sequences were aligned by Blastx to protein databases such as NR (NCBI nonredundant protein), Swiss-Prot, KEGG (Kyoto Encyclopedia of Genes and Genomes) and COG (Cluster of Orthologous Groups) and aligned by Blastn to the nucleotide database NT (*E*-value < 0.00001). In addition, we used Blast2 GO program [58] to obtain GO (Gene Ontology) [41] annotation of unigenes. GO has three ontologies describing molecular function, cellular component and biological process. After that, WEGO software [59] was used to GO functional classification for all unigenes. Finally, with the KEGG database, we could further study the complicated biological behaviors of unigenes and get pathway annotation of them.

### 4.11. Analysis of Differences in Unigene Expression

To predict unigene expression levels in different samples, we calculated the unigene expression using the FPKM (fragments per kb per million fragments) method [60]. This method can eliminate the influence of different gene lengths and sequencing levels, so the calculated unigene expression can be directly used for comparing unigene expression differences between samples. After calculation, differentially expressed genes (DEGs) were detected between two samples by using the method of Audic and Claverie [61]. In our analysis, DEGs should meet the criteria that FDR (false discovery rates) ≤ 0.001 and fold change ≥ 2, then GO functional analysis and KEGG pathway analysis were carried out for DEGs.

In GO functional analysis, all DEGs were mapped to each term of Gene Ontology database (http://www.geneontology.org/) and the gene number of each GO term was counted. After that, hypergeometric test was used to find significantly enriched GO terms in DEGs. In KEGG pathway analysis, significantly enriched metabolic pathways or signal transduction pathways were identified in DEGs.

### 4.12. Quantitative Real-Time PCR (qRT-PCR) Validation

To ensure the libraries’ reliability, we randomly selected 10 DEGs from each library and validated the data by qRT-PCR with three biological replicates. Gene specific primers were designed using Primer Premier 5.0 (Premier Biosoft International, Palo Alto, CA, USA) and the reference gene was the chrysanthemum EF1α (Elongation Factor 1a) gene (Genbank accession number KF305681). The sequences of all primers including those of EF1α were listed in Tables S1 and S2. The amplification with these primers was specific and the efficiency of qPCR was about 100%. qRT-PCR was performed on the Mastercycler ep realplex device (Eppendorf, Hamburg, Germany). Each 20 μL qPCR mixture contained 10 μL SYBR Premix Ex Taq II (Takara), 1 μL forward primer, 1 μL reverse primer, 3 μL ddH_2_O and 5 μL cDNA template. The cDNA was synthesized from 1 µg total RNA with PrimeScript^®^ Reverse Transcriptase (Takara), following the manufacturer’s instructions. The quality of RNA met the criterion that the values of A260/A280 were around 2.0 and the values of A260/A230 were all larger than 2.0. The PCR cycling regime was initially denatured (95 °C/2 min), followed by 40 cycles of 95 °C/10 s, 55 °C/15 s and 72 °C/20 s. A melting curve was followed each assay to confirm the amplicons’ specificity. Relative expression levels of the DEGs were calculated by 2^−ΔΔ*C*t^ method [41,62].

## 5. Conclusions

We performed a series of cellular experiments and RNA-seq to investigate the differences in fertility among progenies from self-pollinated chrysanthemum. Finally, we concluded that seed set of “Q10-33-1①”, “Q10-33-1③” and “Q10-33-1④” were mainly determined by pollen germination percentage, while self-pollinated failure of “Q10-33-1⑩” was determined by pistil receptivity. That is to say, “Q10-33-1⑩” was self-incompatible. Moreover, we guessed both SSI system similar to Brassicaceae SI and S-RNased-based GSI system functioned in chrysanthemum SI. According to abnormal pollen tube in Figure 4H and previous studies [7,63], we came to a conclusion that SSI system played a crucial role. These results will help us to produce self-compatible chrysanthemum cultivars in future, which can further help us to create chrysanthemum pure lines.

## Figures and Tables

**Table 1:** Flowering traits of “Q10-33-1”’s four progenies.

Progeny	Flower Color	Petal Shape	Anthotaxy Diameter (cm)	Flower Disc Diameter (cm)	Length of Ray Floret (cm)	Width of Ray Floret (cm)	The Number of Ray Floret	The Number of Tubiform Floret
“Q10-33-1①”	Pink-white	flat	5.00 ± 0.09 ^b^	1.37 ± 0.04 ^a^	2.57 ± 0.11 ^ab^	0.64 ± 0.02 ^c^	25.60 ± 1.36 ^b^	208.00 ± 7.49 ^b^
“Q10-33-1③”	Pink-white	cochleariform	5.30 ± 0.04 ^a^	1.36 ± 0.02 ^a^	2.72 ± 0.04 ^a^	0.78 ± 0.02 ^b^	28.80 ± 1.16 ^ab^	168.40 ± 4.56 ^c^
“Q10-33-1④”	Yellow	flat	5.31 ± 0.05 ^a^	1.36 ± 0.01 ^a^	2.47 ± 0.05 ^b^	0.86 ± 0.03 ^a^	34.40 ± 2.77 ^a^	202.20 ± 4.66 ^b^
“Q10-33-1⑩”	White	flat	5.30 ± 0.08 ^a^	1.38 ± 0.01 ^a^	2.65 ± 0.03 ^ab^	0.83 ± 0.01 ^a^	32.60 ± 1.21 ^a^	246.80 ± 5.60 ^a^

Values given are mean ± standard error. Values with different superscript indicate significant differences at *p* ≤ 0.05 according to Tukey’s test.

**Table 2:** Pollen germination percentages of “Q10-33-1”s four progenies.

Progeny	Pollen Germination Percentage (%)
“Q10-33-1①”	28.81 ± 1.42 ^b^
“Q10-33-1③”	21.02 ± 1.36 ^c^
“Q10-33-1④”	15.13 ± 1.63 ^d^
“Q10-33-1⑩”	33.60 ± 2.85 ^a^

Values given are mean ± standard error. Values with different superscript indicate significant differences at *p* ≤ 0.05 according to Tukey’s test.

**Table 3:** Percentage of full ovaries at different days after self-pollination in “Q10-33-1”’s four progenies.

Progeny	Days after Self-Pollination			Seed Set (%)
	12 Days	18 Days	30 Days	
	Full Ovary (%)	Full Ovary (%)	Full Ovary (%)	
“Q10-33-1①”	58.68 ± 5.32 ^a^	44.83 ± 3.60 ^a^	42.11 ± 2.41 ^a^	37.23 ± 3.17
“Q10-33-1③”	25.72 ± 5.52 ^b^	20.96 ± 2.20 ^b^	18.46 ± 2.92 ^b^	26.77 ± 2.24
“Q10-33-1④”	22.98 ± 4.40 ^c^	14.64 ± 1.66 ^c^	12.12 ± 3.35 ^c^	7.97 ± 0.93
“Q10-33-1⑩”	0 ^d^	0 ^d^	0 ^d^	0

Values given are mean ± standard error. Values with different superscript indicate significant differences at *p* ≤ 0.05 according to Tukey’s test. Comparisons were made among different progenies. Seed set were quoted from our previous study [23].

**Table 4:** Summary of sequencing reads after filtering in chrysanthemum stigmas.

Sample	Total Raw Reads (Mb)	Total Clean Reads (Mb)	Total Clean Bases (Gb)	Clean Reads Q20 (%)	Clean Reads Q30 (%)	Clean Reads Ratio (%)
3S	55.53	44.52	6.68	97.18	91.68	80.17
10S	55.53	44.19	6.63	97.10	91.54	79.58

Q20: the rate of bases which quality is greater than 20.

**Table 5:** Quality metrics of unigenes in chrysanthemum stigmas.

Sample	Total Number	Total Length	Mean Length	N50	N70	N90	GC (%)
3S	80,646	61,510,907	762	1178	693	315	39.89
10S	91,382	73,442,479	803	1261	744	331	39.36
All-Unigene	113,800	94,376,715	829	1317	782	340	39.40

N50: a weighted median statistic that 50% of the total length is contained in unigenes great than or equal to this value. GC (%): the percentage of G and C bases in all unigenes.

**Table 6:** Summary of sequencing reads after filtering in chrysanthemum anthers.

Sample	Total Raw Reads (Mb)	Total Clean Reads (Mb)	Total Clean Bases (Gb)	Clean Reads Q20 (%)	Clean Reads Q30 (%)	Clean Reads Ratio (%)
3A	55.53	44.25	6.64	97.15	91.60	79.69
10A	55.53	44.37	6.65	97.16	91.68	79.90

Q20: the rate of bases which quality is greater than 20.

**Table 7:** Quality metrics of unigenes in chrysanthemum anthers.

Sample	Total Number	Total Length	Mean Length	N50	N70	N90	GC (%)
3A	77,343	58,453,744	755	1178	689	311	40.11
10A	93,204	71,619,379	768	1195	703	318	39.63
All-Unigene	113,638	91,284,692	803	1276	754	328	39.66

N50: a weighted median statistic that 50% of the total length is contained in unigenes great than or equal to this value. GC (%): the percentage of G and C bases in all unigenes.

**Table 8:** Summary of functional annotation result in chrysanthemum stigmas.

Values	Number	Percentage
Total	113,800	100%
NR-Annotated	57,127	50.20%
NT-Annotated	42,115	37.01%
Swiss-Prot-Annotated	39,577	34.78%
KEGG-Annotated	42,586	37.42%
COG-Annotated	19,476	17.11%
Interpro-Annotated	38,563	33.89%
GO-Annotated	23,423	20.58%
Overall	61,731	54.25%

Overall: the number of unigenes which are annotated with at least one functional database.

**Table 9:** Summary of functional annotation result in chrysanthemum anthers.

Values	Number	Percentage
Total	113,638	100%
NR-Annotated	59,346	52.22%
NT-Annotated	42,785	37.65%
Swiss-Prot-Annotated	40,774	35.88%
KEGG-Annotated	43,970	38.69%
COG-Annotated	20,580	18.11%
Interpro-Annotated	40,653	35.77%
GO-Annotated	24,313	21.40%
Overall	63,517	55.89%

Overall: the number of unigenes which are annotated with at least one functional database.

**Table 10:** Potential stigma *S* genes.

Unigene	10S_FPKM	3S_FPKM	Annotation
CL13054.Contig1_All	2.92	0.16	*S*-receptor-like serine/threonine-protein kinase SD2-5-like
Unigene8775_All	4.3	0.84	*S*-receptor-like serine/threonine-protein kinase At2g19130-like
Unigene12923_All	8.86	3.51	*S*-receptor-like serine/threonine-protein kinase At4g27290
CL49.Contig2_All	4.82	2.05	*S*-receptor-like serine/threonine-protein kinase At2g23200
CL1851.Contig6_All	4.48	1.84	*S*-receptor-like serine/threonine-protein kinase At4g27290
CL2175.Contig4_All	5.54	0.25	*S*-receptor-like serine/threonine-protein kinase B120
CL2302.Contig1_All	5.51	0	*S*-receptor-like serine/threonine-protein kinase B120
CL4068.Contig2_All	3.68	0.31	*S*-receptor-like serine/threonine-protein kinase At2g19130
CL5636.Contig1_All	7.58	1.63	*S*-receptor-like serine/threonine-protein kinase At1g61440
CL13888.Contig1_All	3.91	1.07	*S*-receptor-like serine/threonine-protein kinase At1g11330
CL14810.Contig1_All	5.69	2.65	*S*-receptor-like serine/threonine-protein kinase At1g67520
Unigene6395_All	2.16	0.18	*S*-receptor-like serine/threonine-protein kinase SD2-5
Unigene7043_All	4.68	0.83	*S*-receptor-like serine/threonine-protein kinase At4g27290
Unigene12924_All	4.8	0.52	*S*-receptor-like serine/threonine-protein kinase At4g27290
Unigene21655_All	8.72	3.57	*S*-receptor-like serine/threonine-protein kinase SD1-1
Unigene26414_All	2.7	0	*S*-receptor-like serine/threonine-protein kinase SD1-7
Unigene27592_All	1.85	0.17	*S*-receptor-like serine/threonine-protein kinase At1g11280
Unigene28018_All	2.63	0.29	*S*-receptor-like serine/threonine-protein kinase SD1-1
CL9204.Contig1_All	2.36	0.66	Extracellular ribonuclease LE-like
CL9204.Contig2_All	25.37	8.25	Extracellular ribonuclease LE-like
Unigene9014_All	120.17	65.25	Ribonuclease S-F11-like
Unigene61187_All	1.04	0	Self-incompatibility ribonuclease S5

**Table 11:** Potential pollen *S* genes.

Unigene	10A_FPKM	3A_FPKM	Annotation
CL3238.Contig2_All	3.54	1.1	S3 self-incompatibility locus-linked pollen 3.15 protein
CL10523.Contig1_All	42.99	0.96	*S*-locus-related glycoprotein 1 binding pollen coat protein
CL10523.Contig2_All	16.58	1.79	*S*-locus-related glycoprotein 1 binding pollen coat protein
Unigene8136_All	438.67	87.33	pollen coat-like protein
Unigene10304_All	167.51	5.52	*S*-locus-related glycoprotein 1 binding pollen coat protein
Unigene12281_All	1008.08	217.32	pollen coat-like protein
CL442.Contig8_All	0.73	0	*S*-locus F-Box protein c
CL442.Contig9_All	1.87	1.1	*S*-locus F-Box protein c

## Supplementary Materials

The following are available online at http://www.mdpi.com/1422-0067/19/3/832/s1.
