# Supplementary material for: Investigation of Differences in Fertility among Progenies from Self-Pollinated Chrysanthemum
Source: Int J Mol Sci. 2018 Mar 13;19(3):832. doi: 10.3390/ijms19030832 (PMC5877693; doi:10.3390/ijms19030832)
Supplement: Supplementary file 1 [file ijms-19-00832-s001.zip › Supplementary files/Table S1 Primer sequences of qRT-PCR for stigma transcriptome.doc]

**TABLE S1. Primer sequences of qRT-PCR for stigma transcriptome**

| **Unigene ID** | **Forward primer** | **Reverse primer** |
| --- | --- | --- |
| CL9204.Contig2 | GAAATCAATCTTCTCCAAGCTCTC | TTAGGAAAGACGGGACATTGAA |
| CL6891.Contig2 | AAAGATTGCGATGTGAAGTTGC | AAATACCCCATACACTTACAATCCTT |
| CL1391.Contig5 | CTGAGGCAAGCAATCCATCG | TGCTGGCTGTTTAGGTTTTGG |
| CL2175.Contig2 | TCAGTGTTGGGGAGGGTCTAT | TTCTTCATCTTCTTCGGCAACTA |
| CL7305.Contig7 | AGAGTTATTCACCTTGGGCTGTT | GCTTGGATACATCTCCTCCTCAC |
| CL11828.Contig2 | TGCCCTCCTTGTCATCCTG | CAAGTGGAAAAGAGCTAGAGCAA |
| CL14810.Contig1 | TCCACCTTGTGAGAAAGTGATGA | GTTTGGACGCTCCTCCTCAT |
| Unigene51763 | CATGTCAACATACCAAGCGCA | CACTTGCTATGTTCCAACGACC |
| CL1851.Contig3 | ATGTGTGAATCTTTACGCTCCTC | CGGATGGAAGGTTCTCAAGTT |
| CL2960.Contig3 | GTCGAGTCTCAAGTACTACGATCAAT | AAAATGCTGGTTGTTTAGGTTCA |
| EF1α | CCATTCAAGCGACAGACTCA | TTTTGGTATCTGGTCCTGGAG |
