# Supplementary material for: Investigation of Differences in Fertility among Progenies from Self-Pollinated Chrysanthemum
Source: Int J Mol Sci. 2018 Mar 13;19(3):832. doi: 10.3390/ijms19030832 (PMC5877693; doi:10.3390/ijms19030832)
Supplement: Supplementary file 1 [file ijms-19-00832-s001.zip › Supplementary files/Table S2 Primer sequences of qRT-PCR for anther transcriptome.doc]

**TABLE S2. Primer sequences of qRT-PCR for anther transcriptome**

| **Unigene ID** | **Forward primer** | **Reverse primer** |
| --- | --- | --- |
| CL1824.Contig3 | AAAGAAACTGTAGATGAAAGTGAACC | TGGTAACATTAAAAATAACCCAAGAC |
| CL3143.Contig2 | ATTGCGGTGCATTGTAGTTATTG | CCAATAACTTGCTTGAACTGATGAC |
| CL9992.Contig1 | AACAGCGGGTACATTACTATAGAAGAA | TCTGCCTGGGTTTTGGTCC |
| Unigene3591 | CTTTGTGCATCGTGGGACTG | GATGCTATTTATCAACATTGGGAAC |
| CL4601.Contig3 | TTTGTAGCTAAAAAATTGGGAGAGAG | CGTGGAAATCAAATCATTGTATCAT |
| Unigene10304 | TTCTCATCTAGCTATCCATGTTAAAGT | CATCATTTCTATGTTCAAGATTGTTTAC |
| Unigene12281 | TCATTTCCTCTTTTAGCATCTGGT | AAAAATGGTAAGAGCACGATGAGT |
| CL10523.Contig1 | TGCTCCAACGAAAAATAAGATGT | ACGTGCGTACTTCATACAAAAAAC |
| CL8484.Contig2 | GCTCCCTTGGGCTTTTGTAG | TGAATTACACAAAATAACATGCAGC |
| Unigene42389 | GAAAATCACATAGTCGGTCGCT | CAATGTGCGACACCCAGACT |
| EF1α | CCATTCAAGCGACAGACTCA | TTTTGGTATCTGGTCCTGGAG |
